# Complete genome sequence of *Ekhidna algicida* isolated from Pacific Ocean seawater

**DOI:** 10.1128/mra.01097-25

**Published:** 2026-01-27

**Authors:** Shiri Graff van Creveld, Vaughn Iverson, E. Virginia Armbrust

**Affiliations:** 1 School of Oceanography, University of Washington 7284, Seattle, Washington, USA; California State University San Marcos, San Marcos, California, USA

**Keywords:** filterable microorganisms, bacteria, diatoms, diatom-bacteria interactions, algicidal

## Abstract

*Ekhidna algicida* To15^T^ was isolated from the Tropical Pacific Ocean, based on its algicidal effect on the pelagic diatom *Thalassiosira oceanica*. We report the complete genome sequence, comprising one chromosome for this newly discovered species of “filterable bacteria”, which is pathogenic to diverse diatoms, including diatoms isolated from similar locations.

## ANNOUNCEMENT

Diatoms are the most diverse and abundant group of marine phytoplankton (1), accounting for about 20% of the global primary production (2, 3). Here, we announce the complete genome sequence of *Ekhidna algicida* strain To15^T^ (ATCC TSD-518, DSM 119715T), isolated from surface waters (15 m) in the Pacific Ocean (16°N, 140°W), based on its algicidal effect on the diatom *Thalassiosira oceanica* at 24°C (4). *Ekhidna algicida* To15^T^ is algicidal to diverse diatoms and grows as a pure culture in 50% Marine Broth liquid media. *Ekhidna algicida* To15^T^ was grown in co-culture with *T. oceanica*, in 50-mL filtered autoclaved Puget Sound seawater supplemented by f/2 nutrients (5). Co-culture was grown for 7 days at ~100 μmol photons·m^−2^·s^−1^ 12:12 light:dark cycle at 24°C, then filtered through a 0.2-µm pore-size filter to remove the diatoms and concentrated by centrifugation. DNA was extracted using MasterPure Complete DNA and RNA Purification (MC89010, Biosearch Technologies, UK), following the manufacturer’s instructions for fluid samples.

We used Illumina paired-end (150 bp) and Oxford Nanopore sequences to construct assemblies. Illumina libraries were constructed and sequenced by the Microbial Genome Sequencing Center (Pittsburgh, PA), using the tagmentation-based and PCR-based Illumina DNA Prep kit, custom IDT 10-bp unique dual indices (UDI), and sequenced on an Illumina NovaSeq X Plus sequencer. Demultiplexing, quality control, and adapter trimming were performed with bcl-convert1 (v4.2.4) resulting in 8,220,176 reads (Table 1). The same DNA was used for Nanopore library preparation with the ligation sequencing kit V14 (SQK-LSK114) using Long Fragment Buffer and without size selection. The library was sequenced using a MinION Mk1C and R10.4.1 (FLO-MIN114, FAX02049). Basecalling was performed using Guppy v6.5.7 in fast mode, with a minimum read length of 200 bp and minimum quality score of 8, resulting in a total of 697,564 reads, N_50_ of 25,057 (Table 1).

**TABLE 1:** Data associated with sequencing and assembly

Parameter	Value for *Ekhidna algicida* To15^T^
Genome size	3,967,350 bp
Illumina reads	8,220,176
Nanopore reads	697,564
Nanopore N_50_	25,057
Completeness	95.30%
Contamination	0.90%
Protein coding genes	3,561
Pseudo genes	3
Average protein length	341.76 aa
Coding density	93.29%
G+C	40.38%
% Protein-encoding feature coverage	92.76
% Features that are hypothetical	60.95
Complete rRNA	3
tRNA	35
Secretion systems	Sec, T9SS
Proteases	22
Peptidase	106
Hemolysin	2

Assembly was performed using Flye (v.2.9.1) (6), integrated within the Geneious Prime (v.2024.0.7) software package, with the following parameters: --nano-raw ,--genome-size 10m, --meta flag and --iterations 1. Oxford Nanopore reads were processed, resulting in five contigs with a total assembly length of 3,983,751 bp and a mean coverage of 1,443. The longest contig was circularized by the Flye assembler and contained the draft genome. The four remaining contigs all contained repetitive sequence with low coverage and read quality. The draft genome was polished using the Illumina paired reads. Two rounds of reassembly using Bowtie2 (v.2.4.5) (7) were carried out within the same software package. First, using the Bowtie2 “--sensitive” alignment preset, 8,088,060 out of 8,220,176 reads were successfully aligned to the draft genome. Second, using the “--very-sensitive” preset resulted in 8,101,190 reads aligning to the prior consensus sequence. Both reassembly rounds allowed for insert sizes between 0 and 800 bp. The finished circular 3,967,350 bp genome was automatically annotated by NCBI Prokaryotic Genomes Annotation Pipeline v6.9, resulting in 3,561 predicted genes (Table 1). The genome encodes for type IX secretion system (T9SS, Table 1), often related to pathogenicity in Bacteroidota (8). Potential pathogenic proteins: proteases, peptidases, and hemolysin were detected (Table 1).

## Data Availability

The complete genome sequence of *Ekhidna algicida* To15^T^ has been deposited in GenBank under the accession number ASM5137970v1. All raw reads have been deposited in the NCBI Sequence Read Archive (SRA) under the BioSample accession number SAMN46389320. The complete record is available in the NCBI BioProject database under the accession number PRJNA1214876.
